# On the Archaeal Origins of Eukaryotes and the Challenges of Inferring Phenotype from Genotype

**DOI:** 10.1016/j.tcb.2016.03.009

**Published:** 2016-07

**Authors:** Gautam Dey, Mukund Thattai, Buzz Baum

**Affiliations:** 1 MRC Laboratory for Molecular Cell Biology, University College London, Gower Street, London WC1E 6BT, UK; 2 National Centre for Biological Sciences, TIFR, GKVK, Bellary Road, Bengaluru 560065, India

**Keywords:** *Lokiarchaeum*, evolution, GTPase, eukaryogenesis

## Abstract

If eukaryotes arose through a merger between archaea and bacteria, what did the first true eukaryotic cell look like? A major step toward an answer came with the discovery of *Lokiarchaeum*, an archaeon whose genome encodes small GTPases related to those used by eukaryotes to regulate membrane traffic. Although ‘Loki’ cells have yet to be seen, their existence has prompted the suggestion that the archaeal ancestor of eukaryotes engulfed the future mitochondrion by phagocytosis. We propose instead that the archaeal ancestor was a relatively simple cell, and that eukaryotic cellular organization arose as the result of a gradual transfer of bacterial genes and membranes driven by an ever-closer symbiotic partnership between a bacterium and an archaeon.

## Trends

Eukaryotes are thought to be a product of symbiosis between archaea and bacteria. The recently discovered *Lokiarchaeum* (‘Loki’) encodes more Eukaryotic Signature Proteins (ESPs) than any other archaeon, making it the closest living relative to the putative ancestor of eukaryotes.

*Lokiarchaeum* is the first prokaryote found to encode small GTPases, gelsolin, BAR domains, and longin domains, leading many to suggest that it might be compartmentalized and be capable of membrane trafficking.

Many models for the evolution of eukaryotes invoke an archaeal ancestor that is capable of phagocytosis to explain the entry of the future mitochondrion into the host cell.

Understanding the cell biology of *Lokiarchaeum* will be key to understanding the morphological transitions that characterized the evolution of eukaryotic cellular architecture, but Loki has not yet been cultured or seen.

## A Hunt for the Archaeal Ancestor of Eukaryotes

The internal architecture of all eukaryotic cells is drastically different from that of their distant relatives bacteria and archaea. Most obviously, they differ in size: eukaryotes are thought to have arisen from prokaryotic ancestors, but eukaryotic cells tend to be one to two orders of magnitude larger in mass than prokaryotes. Further, while the cytoplasm of most prokaryotes is bounded by one or two [1] simple membranes, a series of internal membranes divides the cytoplasm of all eukaryotic cells into numerous internal compartments. The dynamic organization of these compartments is regulated by a startling array of regulatory and structural proteins [2], with many layers of molecular machinery working to ensure the controlled distribution of compartments between daughter cells at cell division [3].

Debates about the cellular nature of the last eukaryotic common ancestor (LECA) and the genetic composition of pre-LECA lineages have raged for decades. It is now widely accepted that eukaryotes represent the fruit of a symbiosis between an archaeal host [4] and at least one bacterial lineage [5], the former likely giving rise to the cell proper and the latter giving rise to mitochondria [6]. However, the lack of intermediates that bridge the gap in size and complexity between prokaryotic precursors and eukaryotes has ensured that eukaryogenesis remains one of the most enduring mysteries in modern biology. Recently, however, the falling costs of sequencing have enabled improved metagenomic sampling of diverse environments, leading to a large increase in the diversity of sequenced archaeal genomes. Remarkably, many of these contain sequences homologous to genes that play critical roles in the organization of eukaryotic cells as they grow and divide, which were previously thought to be unique to eukaryotes. These include the replication initiation complex, ubiquitin, and histones, and many of the proteins thought to underpin the dynamic architecture of eukaryotic cells, including actin, tubulin, and ESCRTIII 7, 8, 9. It now seems clear that the bulk of the machinery governing eukaryotic intracellular architecture derives from proteins present in members of the so-called TACK superphylum of archaea [7]. The discovery of *Lokiarchaeum* (‘Loki’), a novel TACK archaeon named for the deep-sea vent near where it was identified through metagenomic sampling [10], has provided strong support for this idea. The Loki composite genome encodes more homologs of Eukaryotic Signature Proteins (ESPs) than any other prokaryotic genome to date, making it an excellent candidate for a representative of the lineage that gave rise to eukaryotes (Box 1).

Interestingly, Loki is also the first bacterial or archaeal genome found to encode large numbers of proteins with clear homology to eukaryotic small GTPases. This has led to a great deal of excitement in the field because, in eukaryotes, these small GTPases plays key functions in the regulation of the cytoskeleton, cell motility, compartment identity, and intracellular trafficking. Moreover, the molecular identity of intracellular trafficking compartments and the specificity of their interactions are tightly coupled to the variety of nonredundant Rab- and Arf-type small GTPases 11, 12, 13. In eukaryotes, the expansion of specific GTPase families through serial gene-duplication events has also been linked to an increase in compartment diversity over evolutionary time 14, 15, 16.

## What Does Loki Look Like?

As a potential living model for the protoeukaryotic cell, we might wonder what Loki looks like. Does it have a rudimentary trafficking system and primitive organelles, as suggested by recent commentaries 17, 18, 19, or might it be a small, structurally simple archaeon with a large complement of regulatory genes? In the first scenario, a Loki-type cell with complex internal organization may have engulfed a bacterial cell leading to late acquisition of mitochondria. Alternatively, in the second scenario, eukaryotic cellular architecture may have emerged gradually through the influx of lipids and lipid metabolic genes from a bacterial partner 6, 20 during a long period of increasing intimacy. In the latter case, eukaryogenesis was a true collaborative venture that relied on structural and information-processing genes from archaea and on lipid metabolism from bacteria. These are drastically different ways of viewing the origins of eukaryote cell architecture, and Loki holds the key to distinguishing between them.

Unfortunately, at present, members of the Lokiarchaeota and their relatives have yet to be isolated, imaged, or cultured. All that is available is a genome sequence. This forces us to ask an age-old question in biology: is it possible to predict phenotype from genotype? Inferring the form and behavior of an organism from genomic information alone is difficult, especially when the gene families of relevance are ancient and their relationships uncertain. This problem is well illustrated for proteins like actin, where a clear correspondence between the six actin homologs in Loki and the actin genes in eukaryotes remains to be established. Additionally, the problem of inferring cell morphology from sequence data is confounded by the nonlinear relationship between genotypic and phenotypic information. For example, small variations in the structure of a monomer of a cytoskeletal protein can lead to dramatic changes in the behavior of the filament polymer and the resulting cellular phenotypes 21, 22. Nevertheless, despite these challenges, some insights about the appearance of Loki can be gained using phylogenetics, bioinformatics, and cell biology as a guide. Following this line of reasoning we argue that Loki is likely to be a structurally simple cell and that the origins of eukaryotic complexity lie elsewhere. We suggest that a partnership between an ancient Loki-like archaeon and a pre-mitochondrial bacterium allowed lokiarchaeal GTPases to combine with bacterial lipid synthesis, enabling the subsequent evolution of quintessentially eukaryotic membrane-bound compartments.

## Loki GTPases and Membranes

In search of clues to resolve this question, we look at the lessons that can be learned from small GTPases. The identification of numerous ‘Ras-like’ (Ras/Rho/Rab/Ran) and ‘Arf-like’ (Arf/Sar) small GTPases, as well as homologs of the atypical vacuolar/lysosomal Rag GTPases [23], was one of the major surprises of the Loki genome. Although the phylogenetic analyses performed thus far provide only modest support for an archaeal origin of the Ras-like, Arf-like, and Rag-like small GTPase subgroups [23], this information has been used to support the argument that Loki is likely to possess intracellular compartments and, perhaps, a primitive form of phagocytosis 17, 18, 19. If true, this finding would be significant because it provides a mechanism by which a Loki-like cell could have engulfed the future mitochondrion. There are, however, problems with this reading of the data. The presence of large numbers of small GTPases in the Loki genome provides strong evidence of ancestry and the capacity for regulatory complexity but does not by itself imply conservation of function. What, then, is the evidence that, like their eukaryotic counterparts, Loki small GTPases regulate membrane dynamics and compartment identity?

In eukaryotes, many small GTPases are physically associated with membranes and this membrane anchoring plays a fundamental role in linking the GTP–GDP cycle to membrane identity, dynamics, and compartmentalization 12, 13. Small GTPases are recruited to membranes through multiple targeting mechanisms. Most commonly this relies on polybasic sequences that provide an electrostatic interaction with the membrane surface, together with the cotranslational or post-translational addition of one or more lipid tails 24, 25. These lipid modifications include *N*-myristoylation (Arf GTPases), palmitoylation (H-Ras), farnesylation (Ras), and geranylgeranylation (Rab and Rho). Farnesylation and geranylgeranylation (collectively known as prenylation) usually rely on the presence of a ‘CAAX’ box (Cys-aliphatic-aliphatic-X) at the carboxyl terminus of target proteins, where the C-terminal amino acid (X) determines whether the protein will be modified by the closely related enzyme farnesyl transferase (FTase) or geranylgeranyltransferase I (GGTase I). Rab proteins are geranylgeranylated at two C-terminal cysteines by GGTase II, with the aid of a Rab escort protein (REP), which provides specificity [24]. While a single geranylgeranyl tag can ensure the stable association of a protein with a membrane, proteins that are farnesylated often require a second signal (e.g., palmitate tag, polybasic charged residue cluster) for membrane binding. Importantly, the enzymes responsible for these key post-translational lipid modifications are encoded by highly conserved, essential genes ubiquitous across eukaryotes [26]; homologs have yet to be identified in prokaryotes.

Until the discovery of Loki, it seemed clear that small GTPases and their ubiquitous lipid-modifying enzymes coevolved. This is no longer the case. Loki has no detectable orthologs of any of the lipid modification enzymes or accessory proteins discussed above (Table 1). An analysis of all 109 putative Loki small GTPase sequences [23] (NCBI) shows that none has a C-terminal CAAX domain, although putative C-terminal interaction sites for GGTase II were identified in two Loki GTPases (Table S1 in the supplemental information online). In addition, the Loki genome ensemble appears to lack homologs of RhoGDI and RhoGDF, the proteins in eukaryotes that act to regulate the association of lipid-modified small GTPases with membranes (GDI masks the lipid moiety enabling it to maintain small GTPases in the cytosol until they are displaced through the action of GDF [27]), again arguing against Loki GTPases being subject to eukaryote-like lipid modifications. Of course, this does not preclude the presence of an alternative mode of lipid modification in Loki. Since protein–lipid and lipid–lipid interactions are strongly dependent on environmental pressure, temperature, and chemical conditions, it is possible that, for example, the tethering of GTPases to archaeal-type membranes present in Loki at 4°C necessitates a different type of chemical modification. The identification of high temperature and/or mesophilic Lokiarchaeota will help to make the role of the environment clearer.

Thus, either Loki has GTPase-regulated compartments but utilizes a currently unknown mode of membrane association or Loki carries a large complement of GTPases not physically associated with membranes that perform diverse regulatory functions like those played by kinases in modern eukaryotes. In this case, small GTPases emerged as a diversified family of non-membrane-associated regulators in archaea that became associated with membranes during the subsequent process of eukaryogenesis. Although we do not currently have access to cell biological data for Loki, there may be ways to test these two ideas. While functional studies in Loki remain a distant dream, clues can be found in the organization of the genome. This is because many genes in bacteria and archaea – including those of Loki – are assembled into operons. These coregulatory units facilitate the coexpression and coinheritance [via horizontal gene transfer (HGT)] of functionally related genes 28, 29. The identification of proteins that lie alongside each of the different GTPases in Loki operons will therefore provide a clue to their subcellular localization and function (e.g., lipid modification enzymes, kinases, actin homologs, membrane proteins). In addition, it may be possible to determine whether Loki cells are likely to possess physically distinct membrane domains like those that characterize eukaryotic compartments, by looking for patterns of amino acid use and hydrophobicity within transmembrane regions of proteins encoded in the composite genome [30]. At present, without such data, it is hard to argue that Loki has a capacity for intracellular trafficking or phagocytosis as seen in eukaryotes. Indeed, specialized phagocytic machinery in eukaryotes does not appear to be ancestral [31]. Note that this does not preclude there being proteins present in the Loki genome that have the capacity to bend, push, or invaginate membranes, since such proteins are a prerequisite for cell division [32] in both archaea and bacteria.

What can be concluded if these investigations fail to support any membrane-associated role of Loki's small GTPases? Perhaps the small GTPases found in the Loki genome function more like the GTPase Ran 33, 34. Ran has been suggested to be the primordial eukaryotic small GTPase, in part because it is highly conserved and is present in a single copy in all eukaryotes known to date 35, 36, 37. Ran GTPase is not known to insert or associate with membranes (although it is lysine acetylated [38]). Intriguingly, Ran controls traffic across compartments that are separated not by a continuous membrane but by large, semipermeable aqueous channels such as the nuclear pore complex and the ciliary base. This is achieved through the establishment of gradients of Ran GTP activity driven by the spatial separation of its activators and inhibitors. For example, the binding of a Ran GEF to chromatin is used to control the shuttling of proteins between the nucleoplasm and cytoplasm [39] and for spindle-pole positioning [40]. Ran is thought to function a similar way to aid the selective accumulation of proteins within cilia 41, 42.

There is a second set of small GTPases that are not subject to lipid modification that is exemplified by the GTPase Sar1 and the atypical GTPases Miro1/2 (together with Rit and RhoBTB). In eukaryotes, these small GTPases carry membrane-insertion domains. In the case of Sar1, which is present in a single copy in most eukaryotic genomes, this serves to induce the budding of membrane from the endoplasmic reticulum (ER) [43]; in the case of Miro1/2, this hydrophobic domain tethers the GTPases to the outer mitochondrial membrane 44, 45, where they regulate mitochondrial activity and dynamics. These further exceptions to the rule in eukaryotes are interesting in that they represent small GTPases that associate with stable organelles, the ER, the nuclear envelope, and mitochondria rather than self-organizing, dynamic cellular compartments like those regulated by Arf and Rabs. Moreover, it has been argued that the acquisition of these ubiquitous eukaryotic compartments – the continuous nuclear envelope and ER and mitochondria – is likely to represent two key steps in eukaryogenesis [46]. Interestingly, two small GTPases in the Loki genome have hydrophobic alpha helices: KKK46087 and KKK46086 (Table S1), suggesting that they may associate with membranes in this way.

Finally, Rag GTPases are not subject to lipid modification, associating with membranes indirectly instead through recruitment by the Ragulator complex [47]. Moreover, their primary function appears to be in growth signaling via mTOR recruitment to the lysosomal/vacuolar surface, not in membrane deformation or the specification of compartment identity. Thus, it would seem most likely that this ancient family of GTPases performs a regulatory function in Loki analogous to its role in eukaryotes.

## A Bacterial Origin for GTPase Lipid Modifications

How, then, could the capacity for lipid modification and membrane anchoring of small GTPases have evolved? Also, why are lipid-modified GTPases found only in eukaryotes?

Both bacteria and archaea widely utilize post-translational protein modifications [48], particularly lipidation (notably, haloarchaeal secreted proteins [49]). Bacterial metabolic pathways [50] commonly utilize the myristate precursors required for *N*-myristoylation, while isoprenoid side chains (the same used for geranylation and farnesylation) are present as components of archaeal cell membranes [51]. Therefore, could the capacity to lipidate GTPases have arisen through an infusion of bacterial genes into the archaeal genome during eukaryogenesis? A few lines of evidence are consistent with this idea. Phylogenetic analyses show that Rab GGTases (and the related REP) are derived from the same ancestral protein as the alpha subunit of farnesyl transferase and GGTase I [52], which is likely to have been constructed from multiple tetratricopeptide repeats, a motif that is widespread in archaea, bacteria, and eukaryotes. By contrast, the shared beta (catalytic) subunit of the prenyltransferases can be assigned to a larger superfamily of enzymes that catalyze reactions involving polyisoprenes, thereby generating cyclized precursors to hopanoids in bacteria and sterols in animals [53]. The bacterial squalene–hopene synthase exhibits structural similarities to the prenylase beta subunits, with concordance in their secondary structure as well as their active site [53]. These findings suggest a plausible bacterial origin for the enzymes responsible for prenylation. The other two types of GTPase modification, *N*-myristoylation and palmitoylation, involve the addition of fatty acid side chains, which again suggests a bacterial origin because fatty acid-derived lipids are dominant membrane components of bacterial and eukaryotic membranes but are rarely found in archaea [54]. Taken together these arguments support the idea that a symbiotic bacterial partner was the source for many of the metabolic precursors and enzymes responsible for fatty acylation.

## Membrane Fission, Fusion, and Deformation in Loki

What about proteins that function directly in membrane deformation? Do these provide us with additional clues about the likely organization of Loki? The membrane-bound compartments that are defining features of all eukaryotic cells are dynamic entities, constantly exchanging material with one another via vesicles while maintaining their unique identities. Maintenance of the vesicle traffic that gives rise to these dynamic structures requires a host of additional molecular machinery beyond GTPases, including proteins that bend membranes and that mediate membrane fission and fusion events [2]. These proteins therefore provide a further test of the idea that Loki has compartments and might be capable of phagocytosis.

First, COPII coats/scaffolds function in eukaryotes to curve membranes at nuclear pores and into vesicle-shaped structures [55] but have yet to be identified in a Loki genome [23]. Second, dynamins mediate a wide range of membrane scission events in eukaryotes [56] but do not appear to be present in Loki [23]. Instead, they have been suggested to have been inherited with the bacterial ancestor of mitochondria [57]. Third, the membrane fusion machinery in eukaryotes, such as the vesicle–vesicle fusion that is mediated by SNAREs and the associated NSF machinery, many of which carry longin domains [58], are conserved across all eukaryotes [59]. The Loki composite genome does encode 41 longin-like domains, some fused to GTPases [23]. However, this finding alone does not indicate a trafficking role, because members of the large and diverse PAS/GAF superfamily (of which longins are a part) are present in all three domains of life and many are known to interact with GTPases [60]. Moreover, longin-domain proteins regulate the activity of Rag GTPases without either being directly associated with a membrane [61]. Loki longins have no detectable trace of a canonical SNARE or coiled-coil domain, and indeed no such domains were found in the Loki genome [23]. Perhaps additional Loki genomes will be required to establish the origin of the SNARE domain and the relationship between Loki longin domain-containing proteins and SNAREs. In summary, these data do not provide good evidence to support the idea that Loki cells have dynamic membrane compartments. Finally, Loki carries many of the genetic hallmarks of a ‘classical’ member of the TACK family of archaea [7], which include much of the machinery underpinning archaeal membrane lipid biochemistry [10]. Archaeal membranes are not amenable to the type of rapid transitions between phases [62] that typify the membrane contortions that underpin vesicle trafficking in eukaryotes. This is likely to be an additional barrier to the generation of compartments that could be overcome only through the acquisition of bacterial-type lipids from a symbiotic partner.

## The Emergence of Compartmentalization: Slow Eukaryogenesis

**Figure 1:**
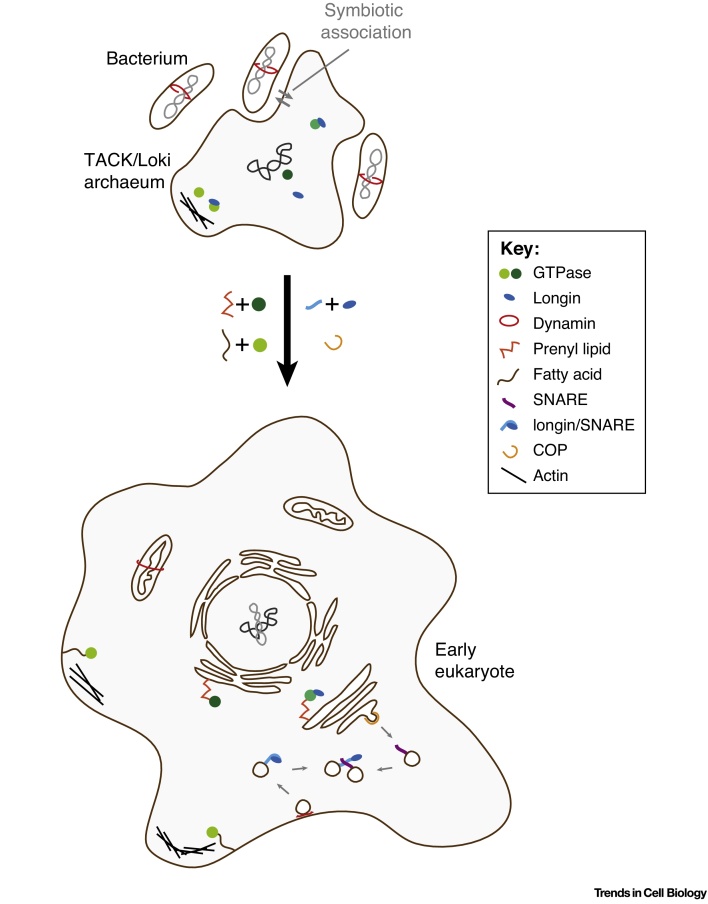
Key Figure: Model for the Evolution of Compartmentalization

Taking all of the above together, any hypothesis of a Loki-like organism being one of the symbiotic partners for eukaryogenesis must contend with the following facts: eukaryotic membrane traffic is tightly regulated by a network of lipid-modified, membrane-associated regulatory GTPases. The Loki genome encodes GTPase families but shows no evidence that these are membrane-associated proteins. Also, bacterial enzymes can perform appropriate lipid modifications but these are missing in Loki. The picture that emerges is of an archaeal host cell that acquired bacterially derived lipids and lipid modification enzymes leading to the association of cytoplasmic GTPase timers with membranes. The ability to label membranes and endow them with distinct chemical properties associated with distinct GTPase homologs sets the stage for the generation of a wealth of membrane-enclosed compartments (Figure 1, Key Figure).

How was the lipid synthesis and modification system transferred from a bacterial donor to an archaeal recipient *in toto*? A sudden switch seems unlikely, as it would disrupt enormous numbers of processes in the host cell that rely on archaeal lipid chemistry. The only alternative is a model in which the transfer occurred in stages. This would require either close and stable contact between donor and recipient or sustained but rare genetic exchange events compounded over large periods of time. In support of the former, evidence suggests that mesophilic environments are conducive to ecological interactions between archaea and bacteria [63]. This arrangement facilitates gene exchange between the two partners. More speculatively, it might also support lipid exchange, perhaps via lipid nanotubes 64, 65, 66. If such exchange were possible, it would produce a cellular membrane environment comprising a unique mix of host-derived archaeal lipids and externally sourced bacterial lipids. Bacterial lipid-associated genes might thus become beneficial to the archaeal recipient and be stably maintained after transfer. It is even possible that the two chemical categories of lipid modification (prenylation and fatty acylation) assisted with the partitioning of GTPases into specific membranes, with the isoprenoid side chains becoming associated with archaeal membrane lipids and the fatty acid side chains with bacterial membrane lipids. If so, this may explain why protein myristoylation recruits diverse proteins like actin and gelsolin to mitochondrial membranes during apoptosis [67], a function that was probably inherited from the alpha-proteobacterial partner [68], and why, for a subset of proteins, post-translational myristoylation dynamically regulates their partitioning between the ER and mitochondrial membrane 69, 70. Conversely, this may also explain why the deprenylation of GTPases such as Rac1 leads to nuclear accumulation [71].

In this model, eukaryogenesis was not a singular event but an ever-closer association between two partner species. As long as gene and lipid exchange could occur, a sustained partnership could lead to the development of a dynamic system of compartments and trafficking in the archaeon. The bacterial cell could have taken up stable residence inside the archaeon at any intermediate stage during the development of the membrane traffic system, ensuring the vertical inheritance of mitochondria.

## Concluding Remarks

We have suggested that, despite encoding numerous small GTPases, Loki lacks much of the machinery required to assemble the equivalent of the eukaryotic vesicle trafficking network. Instead we propose that the archaeal host developed vesicle trafficking capabilities following the acquisition of lipid metabolic genes and lipids from bacteria. For this, the archaeon would need to be in close, stable symbiotic contact with one or more bacterial partners.

Loki is so far unique among archaea in having a large number of highly conserved actin and actin-like proteins, proteins with homology to gelsolin, representatives of all three ESCRT complexes, and a putative BAR domain protein. However, these ESPs most likely function within a typical TACK family archaeal cellular milieu. It follows that we should look to other TACK archaea as a guide to potential function in addition to eukaryotes. For example, the actin homolog crenactin [72] is thought to function in the TACK-related archaeal cell *Pyrobaculum calidifontis* to provide cells with a stable rod-shaped form without conferring the capacity to dynamically change shape. Given its larger complement of actin-like and potential gelsolin-like regulators, Loki may then be capable of assuming different forms. However, given the recent discovery of actin's involvement in nuclear functions 73, 74, actin homologs could function to regulate gene expression in Loki. In a similar vein, since all cells have to divide, it should be no surprise that all bacteria and archaea encode machinery that enables their membranes to undergo regulated or unregulated [75] membrane scission. In many archaea, especially in the TACK group related to Loki, the machinery involved in the scission event that leads to the completion of cell division is ESCRTIII [32], as in eukaryotic cells. Thus it is likely that, in Loki, ESCRTIII does this job: inducing a change in membrane topology that is as old as cellular life itself.

More generally, it is hard to deduce cell topology from gene homologies alone. While we wait impatiently for the first view of a Loki cell, this fact underscores the importance of studying the cell biology of archaea (see Outstanding Questions). Until a Loki strain has been cultured, this effort should focus on the study of TACK family archaea that can be cultured and easily genetically manipulated [76]. This, we suggest, will provide the community with a molecular understanding of the functions of specific TACK regulatory modules, which is the only way to accurately assess the phenotypic significance of Loki ESPs and to understand how its distant ancestor might have been ‘primed’ for the dramatic sequence of events that led to the emergence of eukaryotes.

Outstanding Questions Can we devise methods to infer cell shape and organization from the genome of a cell we have never seen?

Will an analysis of putative operons help reveal likely targets of the Loki small GTPases?

While the community attempts to isolate and culture *Lokiarchaeum*, what can we learn about fundamental archaeal cell biology by studying conserved regulatory modules in other TACK archaea, like *Sulfolobus*?

Can we use the properties of archaeal transmembrane proteins to predict cellular membrane properties and infer the presence or absence of distinct subcellular compartments?

Will phylogenies of the *Lokiarchaeum* actins help resolve the ancestry of these protein families and could ancestral reconstruction and expressing proteins in archaeal or eukaryotic model systems help specify their cellular roles?

Will other core components of the eukaryotic membrane-trafficking machinery be found in future genomes of other Loki species or closely related TACK archaea?

Could we use the presence of Rag GTPase homologs in the Loki genome as a handle to investigate archaeal nutrient-sensing and homeostasis pathways?

## Figures and Tables

**Table 1:** Phylogenetic Distribution of Membrane-Trafficking Building Blocks^a^

	Bacteria	TACK Archaea	*Lokiarchaeum*	Eukaryotes	Refs
Small GTPases	−	−	Present	Present	10, 23
CAAX domains	−	−	−	Present	
Prenyltransferases	−	−	−	Present	
Fatty acid transferases	−	−	−	Present	
GDI/GDF/REP/accessory	−	−	−	Present	
Longin/Roadblock	Present	Present	Present	Present	23, 60
SNARE	−	−	−	Present	
Coat proteins	−	−	−	Present	
Dynamins	Present	−	−	Present	[57]
Actin/actin-like proteins	Present	Present	Present	Present	10, 22, 72

a ‘Present’ indicates that a putative or confirmed protein ortholog (or orthologous group/orthologous domain) has been identified in one or more representative species within each column.

## Box 1 The Lokiarchaeum genome

*Lokiarchaeum* was discovered through a metagenomic analysis of marine sediment sampled some distance from an active vent system named Loki's Castle [10] in the Arctic Mid-Ocean Ridge. 16S rRNA sequencing led to the identification of previously unidentified sequences belonging to a deep-branching TACK clade and further refinement produced a composite genome encoding 5381 putative genes given the name ‘*Lokiarchaeum*’. The first analysis of this remarkable ensemble genome identified 92 putative small Ras-like superfamily GTPases, six actin genes, a strikingly eukaryote-like ribosome, and clearly detectable ESCRTIII, ESCRTI, and ESCRT0 complexes. Intriguingly, longin-like proteins and a putative BAR domain protein, whose homologs play important roles in the regulation of eukaryotic cell shape and membrane organization 59, 77, were also identified. A recent sensitive reexamination of the *Lokiarchaeum* genome [23] has identified a further 17 small GTPases, bringing the total to 109 (including some with homology to eukaryotic Rag GTPases). This analysis also revealed the presence of 38 Roadblock domains, a subset of which appears fused to Ras-like and Rag-like small GTPases, and a RLC7 dynein homolog. Finally, additional longin/longin-like domains were identified, so 41 have now been identified in total. Again, intriguingly, five of these were found fused to lokiarchaeal Arf-like small GTPases. Collectively these data support the idea that, despite the intervening events, which include the acquisition of mitochondria, a member of this or a closely related archaeal lineage gave rise to the eukaryotic cell through sequential rounds of growth and division.
