# Supplementary material for: On the Archaeal Origins of Eukaryotes and the Challenges of Inferring Phenotype from Genotype
Source: Trends Cell Biol. 2016 Jul;26(7):476–85. doi: 10.1016/j.tcb.2016.03.009 (PMC4917890; doi:10.1016/j.tcb.2016.03.009)
Supplement: Supplementary file 1 [file mmc1.docx]

*Supplementary Material*

On the Archaeal Origins of Eukaryotes and the Challenges of Inferring Phenotype from Genotype

Gautam Dey^*1^, Mukund Thattai^2^ and Buzz Baum^*1^

^1^MRC Lab for Molecular Cell Biology, UCL, Gower Street, London WC1E 6BT, UK

^2^National Centre for Biological Sciences, TIFR, GKVK, Bellary Road, Bengaluru 560065, India

*Corresponding authors*: Baum, B. ([b.baum@ucl.ac.uk](mailto:b.baum@ucl.ac.uk)); Dey, G. ([g.dey@ucl.ac.uk](mailto:g.dey@ucl.ac.uk))

Supplementary Methods

Analysis of putative Lokiarchaeum small GTPases: transmembrane domains and prenylation motifs

Previous analyses [1,2] have identified 109 small GTPases in the Lokiarchaeum composite genome. The amino acid sequences of the 109 predicted proteins were downloaded in FASTA format from the NCBI Protein database (<http://www.ncbi.nlm.nih.gov/protein>; Lokiarchaeum sp. GC14_75; latest download March 15, 2016) using custom MATLAB scripts (Bioinformatics Toolbox). These sequences were submitted to the following online servers using the default search parameters (in the absence of specifc parameters optimized for archaeal genomes).

Transmembrane domain prediction

1. TMHMM 2.0[3] (<http://www.cbs.dtu.dk/services/TMHMM/>)
2. Phobius[4] (<http://phobius.sbc.su.se/index.html>)

Prenylation sites

1. PrePS[5] (<http://mendel.imp.ac.at/PrePS/>)

Protein sequences containing putative transmembrane domains were submitted to a third server CCTOP[6] ([http://cctop.enzim.ttk.mta.hu](http://cctop.enzim.ttk.mta.hu/)) that aggregates results from different prediction methods, in order to extract consensus start and stop sites for transmembrane domains.

The results of these analyses (as of March 15, 2016) are presented in Table S1.

Table S1: Transmembrane and Prenylation Site Analysis of 109 Loki Small GTPases

For each small GTPase, a negative result is indicated by a dash; if transmembrane domains were identified (green highlight), the start and stop positions of each domain are listed; if prenylation sites were identified, the type of prenylation site is listed (red highlight). PFAM annotations and eukaryotic best matches taken from [2].

| **No.** | **Name** | **GI** | **Accession** | **PFAM** | **Match** | **TMMHMM** | **Phobius** | **Prenylation** | **CCTOP** | **Ref.** |
| --- | --- | --- | --- | --- | --- | --- | --- | --- | --- | --- |
| 1 | Lokiarch_19980 | 816392523 | KKK44255.1 | Arf | Arl11 | - | - | - | - | [1],[2] |
| 2 | Lokiarch_01140 | 816395093 | KKK46455.1 | Arf | - | - | - | - | - | [1],[2] |
| 3 | Lokiarch_51460 | 816388158 | KKK40445.1 | Gtr1_RagA | - | - | - | - | - | [2] |
| 4 | Lokiarch_03700 | 816394752 | KKK46158.1 | Gtr1_RagA | - | - | - | - | - | [2] |
| 5 | Lokiarch_15960 | 816393056 | KKK44706.1 | Arf | - | - | - | - | - | [1],[2] |
| 6 | Lokiarch_05490 | 816394522 | KKK45962.1 | Arf | - | - | - | - | - | [1],[2] |
| 7 | Lokiarch_44040 | 816389146 | KKK41308.1 | Arf | - | - | - | - | - | [1],[2] |
| 8 | Lokiarch_53730 | 816387817 | KKK40160.1 | Arf | Arl3 | - | - | - | - | [1],[2] |
| 9 | Lokiarch_10070 | 816393866 | KKK45400.1 | Arf | - | - | - | - | - | [1],[2] |
| 10 | Lokiarch_05440 | 816394517 | KKK45957.1 | Arf | - | - | - | - | - | [1],[2] |
| 11 | Lokiarch_06040 | 816394423 | KKK45873.1 | Arf | - | - | - | - | - | [1],[2] |
| 12 | Lokiarch_28560 | 816391393 | KKK43261.1 | Arf | Arf | - | - | - | - | [1],[2] |
| 13 | Lokiarch_18170 | 816392730 | KKK44436.1 | Gtr1_RagA | - | - | - | - | - | [2] |
| 14 | Lokiarch_50350 | 816388223 | KKK40499.1 | Arf | - | - | - | - | - | [1],[2] |
| 15 | Lokiarch_38370 | 816389939 | KKK41997.1 | Gtr1_RagA | - | - | - | - | - | [2] |
| 16 | Lokiarch_42960 | 816389296 | KKK41444.1 | Gtr1_RagA | - | - | - | - | - | [2] |
| 17 | Lokiarch_42900 | 816389290 | KKK41438.1 | Gtr1_RagA | - | - | - | - | - | [2] |
| 18 | Lokiarch_15440 | 816393126 | KKK44766.1 | Gtr1_RagA | - | - | - | - | - | [2] |
| 19 | Lokiarch_10260 | 816393828 | KKK45368.1 | Gtr1_RagA | - | - | - | - | - | [2] |
| 20 | Lokiarch_02630 | 816394898 | KKK46284.1 | Gtr1_RagA | - | - | - | - | - | [2] |
| 21 | Lokiarch_43010 | 816389301 | KKK41449.1 | Arf | - | - | - | - | - | [1],[2] |
| 22 | Lokiarch_35420 | 816390359 | KKK42365.1 | Gtr1_RagA | Arl5 | - | - | - | - | [2] |
| 23 | Lokiarch_09660 | 816393970 | KKK45486.1 | Gtr1_RagA | - | - | - | - | - | [2] |
| 24 | Lokiarch_45120 | 816389010 | KKK41196.1 | Arf | - | - | - | - | - | [1],[2] |
| 25 | Lokiarch_04330 | 816394656 | KKK46072.1 | Gtr1_RagA | - | - | - | - | - | [2] |
| 26 | Lokiarch_11040 | 816393734 | KKK45286.1 | Arf | - | - | - | - | - | [1],[2] |
| 27 | Lokiarch_12680 | 816393513 | KKK45093.1 | Gtr1_RagA | Rag | - | - | - | - | [2] |
| 28 | Lokiarch_51600 | 816388131 | KKK40424.1 | Gtr1_RagA | Rag | - | - | - | - | [2] |
| 29 | Lokiarch_50330 | 816388221 | KKK40497.1 | Arf | - | - | - | - | - | [1],[2] |
| 30 | Lokiarch_35740 | 816390321 | KKK42335.1 | Arf | - | - | - | - | - | [1],[2] |
| 31 | Lokiarch_09000 | 816394015 | KKK45525.1 | Arf | - | - | - | - | - | [1],[2] |
| 32 | Lokiarch_17970 | 816392763 | KKK44463.1 | Gtr1_RagA | Rag | - | - | - | - | [2] |
| 33 | Lokiarch_42280 | 816389370 | KKK41504.1 | Arf | - | - | - | - | - | [1],[2] |
| 34 | Lokiarch_46160 | 816388862 | KKK41062.1 | Arf | - | - | - | - | - | [1],[2] |
| 35 | Lokiarch_50090 | 816388339 | KKK40609.1 | Gtr1_RagA | - | - | - | - | - | [2] |
| 36 | Lokiarch_20230 | 816392473 | KKK44209.1 | Arf | - | - | - | - | - | [1],[2] |
| 37 | Lokiarch_38460 | 816389948 | KKK42006.1 | Gtr1_RagA | - | - | - | - | - | [2] |
| 38 | Lokiarch_53190 | 816387894 | KKK40223.1 | Ras | - | - | - | GGT2 C-term site | - | [1],[2] |
| 39 | Lokiarch_36830 | 816390157 | KKK42189.1 | Ras | - | - | - | - | - | [1],[2] |
| 40 | Lokiarch_31810 | 816390903 | KKK42845.1 | Ras | - | - | - | - | - | [1],[2] |
| 41 | Lokiarch_31850 | 816390907 | KKK42849.1 | Ras | Rab14 | - | - | - | - | [1],[2] |
| 42 | Lokiarch_00500 | 816395189 | KKK46541.1 | Ras | - | - | - | - | - | [1],[2] |
| 43 | Lokiarch_02350 | 816394909 | KKK46293.1 | Ras | Rab6 | - | - | - | - | [1],[2] |
| 44 | Lokiarch_22470 | 816392199 | KKK43967.1 | Ras | - | - | - | - | - | [1],[2] |
| 45 | Lokiarch_31930 | 816390915 | KKK42857.1 | Ras | - | - | - | - | - | [1],[2] |
| 46 | Lokiarch_36400 | 816390237 | KKK42259.1 | Ras | Rab14 | - | - | - | - | [1],[2] |
| 47 | Lokiarch_52350 | 816388032 | KKK40341.1 | Ras | - | - | - | - | - | [1],[2] |
| 48 | Lokiarch_15930 | 816393053 | KKK44703.1 | Ras | Rab22 | - | - | - | - | [1],[2] |
| 49 | Lokiarch_45790 | 816388895 | KKK41091.1 | Ras | - | - | - | - | - | [1],[2] |
| 50 | Lokiarch_27490 | 816391517 | KKK43373.1 | Ras | - | - | - | - | - | [1],[2] |
| 51 | Lokiarch_47920 | 816388615 | KKK40851.1 | Ras | Rab8 | - | - | - | - | [1],[2] |
| 52 | Lokiarch_21790 | 816392272 | KKK44032.1 | Ras | - | - | - | - | - | [1],[2] |
| 53 | Lokiarch_44790 | 816389041 | KKK41219.1 | Ras | - | - | - | - | - | [1],[2] |
| 54 | Lokiarch_31830 | 816390905 | KKK42847.1 | Ras | - | - | - | - | - | [1],[2] |
| 55 | Lokiarch_30440 | 816391121 | KKK43031.1 | Ras | - | - | - | - | - | [1],[2] |
| 56 | Lokiarch_18250 | 816392738 | KKK44444.1 | Ras | Rab13 | - | - | - | - | [1],[2] |
| 57 | Lokiarch_18970 | 816392623 | KKK44343.1 | Ras | Rab43 | - | - | - | - | [1],[2] |
| 58 | Lokiarch_01650 | 816395040 | KKK46406.1 | Ras | - | - | - | - | - | [1],[2] |
| 59 | Lokiarch_10880 | 816393749 | KKK45299.1 | Ras | - | - | - | - | - | [1],[2] |
| 60 | Lokiarch_12240 | 816393572 | KKK45140.1 | Ras | - | - | - | - | - | [1],[2] |
| 61 | Lokiarch_33450 | 816390673 | KKK42647.1 | Ras | - | - | - | - | - | [1],[2] |
| 62 | Lokiarch_38560 | 816389915 | KKK41975.1 | Ras | Rab4 | - | - | - | - | [1],[2] |
| 63 | Lokiarch_44980 | 816388996 | KKK41182.1 | Ras | - | - | - | - | - | [1],[2] |
| 64 | Lokiarch_38550 | 816389914 | KKK41974.1 | Ras | - | - | - | - | - | [1],[2] |
| 65 | Lokiarch_39550 | 816389775 | KKK41853.1 | Ras | Rab23 | - | - | - | - | [1],[2] |
| 66 | Lokiarch_01690 | 816395044 | KKK46410.1 | Ras | Rab14 | - | - | - | - | [1],[2] |
| 67 | Lokiarch_48070 | 816388593 | KKK40831.1 | Ras | - | - | - | - | - | [1],[2] |
| 68 | Lokiarch_04620 | 816394637 | KKK46057.1 | Ras | - | - | - | - | - | [1],[2] |
| 69 | Lokiarch_49520 | 816388410 | KKK40672.1 | Ras | - | - | - | - | - | [1],[2] |
| 70 | Lokiarch_51330 | 816388168 | KKK40453.1 | Robl_LC7 | - | - | - | - | - | [1],[2] |
| 71 | Lokiarch_45420 | 816388963 | KKK41155.1 | Ras | - | - | - | - | - | [1],[2] |
| 72 | Lokiarch_33970 | 816390576 | KKK42560.1 | Ras | Rab5 | - | - | - | - | [1],[2] |
| 73 | Lokiarch_17900 | 816392798 | KKK44492.1 | Ras | Rab30 | - | - | - | - | [1],[2] |
| 74 | Lokiarch_12880 | 816393497 | KKK45081.1 | Ras | Rab13 | - | - | - | - | [1],[2] |
| 75 | Lokiarch_33770 | 816390629 | KKK42609.1 | Robl_LC7 | - | - | - | - | - | [1],[2] |
| 76 | Lokiarch_51340 | 816388169 | KKK40454.1 | Ras | Rab5 | - | - | - | - | [1],[2] |
| 77 | Lokiarch_35450 | 816390362 | KKK42368.1 | Ras | - | - | - | - | - | [1],[2] |
| 78 | Lokiarch_30390 | 816391116 | KKK43026.1 | Ras | - | - | - | - | - | [1],[2] |
| 79 | Lokiarch_02670 | 816394902 | KKK46288.1 | Ras | Rab23 | - | - | - | - | [1],[2] |
| 80 | Lokiarch_33750 | 816390627 | KKK42607.1 | Ras | Rab14 | - | - | - | - | [1],[2] |
| 81 | Lokiarch_45850 | 816388901 | KKK41097.1 | Ras | - | - | - | - | - | [1],[2] |
| 82 | Lokiarch_51370 | 816388172 | KKK40457.1 | Ras | - | - | - | - | - | [1],[2] |
| 83 | Lokiarch_32470 | 816390878 | KKK42824.1 | Robl_LC7 | Rab39 | - | - | - | - | [1],[2] |
| 84 | Lokiarch_04160 | 816394696 | KKK46108.1 | Ras | Rab2 | - | - | GGT2 C-term site | - | [1],[2] |
| 85 | Lokiarch_34920 | 816390478 | KKK42474.1 | Ras | Rab33 | - | - | - | - | [1],[2] |
| 86 | Lokiarch_04480 | 816394671 | KKK46087.1 | Ras | - | 2 TM | 2 TM | - | 244-264, 286-310 | [1],[2] |
| 87 | Lokiarch_53220 | 816387897 | KKK40226.1 | Ras | - | - | - | - | - | [1],[2] |
| 88 | Lokiarch_31960 | 816390918 | KKK42860.1 | Ras | Rab23 | - | - | - | - | [1],[2] |
| 89 | Lokiarch_21100 | 816392368 | KKK44114.1 | Ras | Rab11 | - | - | - | - | [1],[2] |
| 90 | Lokiarch_04210 | 816394701 | KKK46113.1 | Ras | - | - | - | - | - | [1],[2] |
| 91 | Lokiarch_39540 | 816389774 | KKK41852.1 | Ras | - | - | - | - | - | [1],[2] |
| 92 | Lokiarch_15320 | 816393137 | KKK44775.1 | Ras | Rab23 | - | - | - | - | [1],[2] |
| 93 | Lokiarch_37110 | 816390119 | KKK42155.1 | Ras | Rab7 | - | - | - | - | [1],[2] |
| 94 | Lokiarch_21460 | 816392314 | KKK44068.1 | Ras | - | - | - | - | - | [1],[2] |
| 95 | Lokiarch_12180 | 816393566 | KKK45134.1 | Ras | - | - | - | - | - | [1],[2] |
| 96 | Lokiarch_11830 | 816393636 | KKK45196.1 | Ras | - | - | - | - | - | [1],[2] |
| 97 | Lokiarch_52000 | 816388066 | KKK40369.1 | Ras | - | - | - | - | - | [1],[2] |
| 98 | Lokiarch_09250 | 816394000 | KKK45512.1 | Ras | - | - | - | - | - | [1],[2] |
| 99 | Lokiarch_18530 | 816392696 | KKK44406.1 | Ras | Rab2 | - | - | - | - | [1],[2] |
| 100 | Lokiarch_26330 | 816391658 | KKK43498.1 | Ras | Rab11 | - | - | - | - | [1],[2] |
| 101 | Lokiarch_09940 | 816393897 | KKK45421.1 | Ras | Rab11 | - | - | - | - | [1],[2] |
| 102 | Lokiarch_04470 | 816394670 | KKK46086.1 | Ras | Rab11 | 2 TM | 2 TM | - | 243-260, 281-301 | [1],[2] |
| 103 | Lokiarch_14020 | 816393331 | KKK44939.1 | Ras | - | - | - | - | - | [1],[2] |
| 104 | Lokiarch_26690 | 816391611 | KKK43455.1 | Ras | - | - | - | - | - | [1],[2] |
| 105 | Lokiarch_26700 | 816391612 | KKK43456.1 | Ras | - | - | - | - | - | [1],[2] |
| 106 | Lokiarch_17300 | 816392870 | KKK44552.1 | Ras | - | - | - | - | - | [1],[2] |
| 107 | Lokiarch_04170 | 816394697 | KKK46109.1 | Ras | - | - | - | - | - | [1],[2] |
| 108 | Lokiarch_01230 | 816395102 | KKK46464.1 | Ras | - | - | - | - | - | [1],[2] |
| 109 | Lokiarch_17150 | 816392899 | KKK44575.1 | Ras | - | - | - | - | - | [1],[2] |

Supplementary References

S1 Spang, A. *et al.* (2015) Complex archaea that bridge the gap between prokaryotes and eukaryotes. *Nature* 521, 173–179

S2 Klinger, C.M. *et al.* (2016) Tracing the archaeal origins of eukaryotic membrane-trafficking system building blocks. *Mol. Biol. Evol.* DOI: 10.1093/molbev/msw034

S3 Krogh, A. *et al.* (2001) Predicting transmembrane protein topology with a hidden Markov model: application to complete genomes. *J. Mol. Biol.* 305, 567–80

S4 Käll, L. *et al.* (2007) Advantages of combined transmembrane topology and signal peptide prediction--the Phobius web server. *Nucleic Acids Res.* 35, W429–32

S5 Maurer-Stroh, S. and Eisenhaber, F. (2005) Refinement and prediction of protein prenylation motifs. *Genome Biol.* 6, R55

S6 Dobson, L. *et al.* (2015) CCTOP: a Consensus Constrained TOPology prediction web server. *Nucleic Acids Res.* 43, W408–12
